# The Unmet Need for Interpreting Provision in UK Primary Care

**DOI:** 10.1371/journal.pone.0020837

**Published:** 2011-06-13

**Authors:** Paramjit S. Gill, Jacqueline Beavan, Melanie Calvert, Nick Freemantle

**Affiliations:** 1 Primary Care Clinical Sciences, School of Health and Population Sciences, University of Birmingham, Birmingham, United Kingdom; 2 Primary Care and Population Health, University College London, London, United Kingdom; Leicester University, United Kingdom

## Abstract

**Background:**

With increasing globalisation, the challenges of providing accessible and safe healthcare to all are great. Studies show that there are substantial numbers of people who are not fluent in English to a level where they can make best use of health services. We examined how health professionals manage language barriers in a consultation.

**Methods and Findings:**

This was a cross-sectional study in 41 UK general practices . Health professionals completed a proforma for a randomly allocated consultation session.

Seventy-seven (63%) practitioners responded, from 41(59%) practices. From 1008 consultations, 555 involved patients who did not have English as a first language; 710 took place in English; 222 were in other languages, the practitioner either communicating with the patient in their own language/using an alternative language. Seven consultations were in a mixture of English/patient's own language. Patients' first languages numbered 37 (apart from English), in contrast to health practitioners, who declared at least a basic level of proficiency in 22 languages other than English. The practitioner's reported proficiency in the language used was at a basic level in 24 consultations, whereas in 21, they reported having no proficiency at all. In 57 consultations, a relative/friend interpreted and in 6, a bilingual member of staff/community worker was used. Only in 6 cases was a professional interpreter booked. The main limitation was that only one random session was selected and assessment of patient/professional fluency in English was subjective.

**Conclusions:**

It would appear that professional interpreters are under-used in relation to the need for them, with bilingual staff/family and friends being used commonly. In many cases where the patient spoke little/no English, the practitioner consulted in the patient's language but this approach was also used where reported practitioner proficiency was low. Further research in different setting is needed to substantiate these findings.

## Introduction

Overcoming language barriers to health care is a global challenge [1]–[4]. In the US about 22 million residents are unable to speak English fluently, with over half of these non-English speakers speaking Spanish and 15 million people speaking 24 different languages [5]. In urban Australia language services are required in up to 100 different languages reflecting enormous linguistic diversity [6]. The UK is a diverse society with 7.9% of the population from the Black and minority ethnic groups [7]. This is a heterogeneous group with different migration and settlement patterns, culture, religion, and languages spoken. Recent research identifying more than 300 languages, excluding dialects, spoken by children at home indicates that London may be the most linguistically diverse city in the world [8]. Indeed within one health authority, the Heart of Birmingham Teaching Primary Care Trust (HOBtPCT), the Black and minority ethnic communities are in the majority comprising 71% of the population in the 2001 Census [9]. However no national data exist on the number of non-English speakers in England and Gill et al [10] have estimated that there are nearly 400,000 adults from the four main minority ethnic communities (i.e. Indian, Pakistani, Bangladeshi and Chinese) who have a need for interpreting. At the health authority level the number of non-English speakers is not known though the Birmingham Integrated Language and Communication Support Service provided interpreters for 30,000 consultations at a cost of over £1,000,000 in 2007/8 and this is expected to rise substantially over the next few years with new migrants from the expanded European Union.

It is obvious that high-quality medical care requires effective communication between patient and health professional [11]. The complexity of linguistic diversity is illustrated by a report that found that many doctors working in primary care are themselves not native English speakers and communicate with their patients, originally from the Indian subcontinent, in one of a range of Asian languages [12]. In fact, the majority of this ‘translation need’ has, and continues to be met by the many overseas trained family medicine doctors who are due to retire imminently [13] and demand for interpreting may rise. An added benefit of these overseas trained family medicine professionals is the shared understanding and knowledge of health beliefs and expectations from health care professionals [14].

When faced with English-speaking health professionals, use of informal interpreters such as family members is common although this may be problematic when faced with embarrassing issues or when the informal interpreter's language skills are poor [15]. While even good quality professional interpreting will not completely remove the language barrier, effective communication can be achieved and has been shown to lead to improved care [16] comparable to that of English speakers [17].

The aim of this study was to examine how family medicine professional manage language barriers in consultations. Specific objectives were to: document the number of general practice consultations occurring in a language other than English, document the use of interpreting services and model the need for interpreting within the health authority and potential implications for commissioning of interpreting services the UK.

## Methods

### Study Population

All 70 primary care centres in HOBtPCT were invited to participate in the study and were given relevant information at a health authority education session. Information was also given to Practice Managers who were present at this session, as they were perceived as being important in the process of co-ordinating participation and the collection of data. This was followed up by the delivery by hand of information packs and supporting documentation to Practice Managers the week before their practice was due to participate.

### Survey content

Each primary care centre was randomly allocated a session in a given week in June 2009 when family medicine and advanced nurse practitioners were asked to complete details on a consultation record sheet for each patient they saw in that session. The survey instrument was devised by the investigators and included patient demographic characteristics, the primary language of the patient, the patient's proficiency in English (as subjectively assessed by the practitioner), the language used in the consultation and the strategy employed where the primary language was not English. Practitioners were also asked to give details of their own proficiency in languages other than English and their job title. (see Appendix S1). The survey instrument was pilot tested for clarity and use in a neighbouring authority.

### Analysis

Analyses were performed using SAS V9.2 (SAS Institute). The characteristics of participants and practitioners were described with frequency for categorical measures and median and interquartile range for continuous measures.

Prognostic modelling techniques were applied to examine whether the characteristics of practitioners or patients predict the need for interpreting services, and the extent of the unmet need (as judged by the practitioner) for interpretation. Potential predictors included the ethnicity of practitioners and patients, the gender of practitioners and patients, number of principals and practice deprivation index. Responses were dichotomised so that ‘None’ or ‘Basic’ were classed as inadequate language skills and ‘Moderately Well’ and ‘Highly Proficient’ were classed as adequate language skills. Where the language of consultation was English, it was assumed that the language skills of the practitioner were adequate. Where no proficiency was described, it was assumed that practitioner skills were inadequate. Where an interpreter was used, practitioner language skills were assessed for the first language of the patient, regardless of the actual language of consultation.

Univariate mixed models were developed, with a logistic link function and binomial/Gaussian error, each including the explanatory variable of interest and including GPs as random effects. Where multiple explanatory variables demonstrated associations with the response variable at a significance level P<.05, a parsimonious multivariable model would be developed using a stepwise model reduction procedure.

The proportion of patients from different ethnic backgrounds requiring an interpreter was used in conjunction with health authority level estimates of ethnicity to model health authority levels of interpreter requirements. Finally, the costs associated with such provision were estimated.

## Results

### Practitioner Characteristics

A total of 122 family medicine practitioners were eligible to participate on the dates allocated to their centres. Of these 77 took part (73 doctors and 4 advanced nurse practitioners) giving a response rate of 63%. 41 out of a possible 70 practices (59%) participated in the study and there were 1008 patient consultations. At the time the study took place, the UK was in the midst of a “swine flu” pandemic first large school outbreak in this health authority, and this may have contributed to a lower response rate than may otherwise have occurred [18].

Most of the responders (n = 56) were family medicine professionals (Table 1). There were more male than female practitioners and the majority of practitioners were from South Asian backgrounds. Most of the participating practitioners in the survey qualified outside the UK, mainly in the Indian sub-continent.

**Table 1:** Demographic characteristics of practitioners.

Practitioner grade	n	%
General Practitioner Principal	36	46.8
Salaried General Practitioner	20	26.0
Locum General Practitioner	4	5.2
*General Practice Trainee	8	11.2
*Foundation Year 2 General Practitioner	2	2.6
Advanced Nurse Practitioner	4	5.2
Not recorded	2	2.6
**Gender**		
Female	30	39.0
Male	45	58.4
Not specified	2	2.6
**Age, median (IQR) n = 68**	48	37.5 to 59.5
**Ethnicity**		
South Asian	59	76.6
White	9	11.7
Arab	3	3.9
Not Stated	6	7.8
**Country of Qualification**		
UK	30	39.0
India	28	36.4
Pakistan	6	7.8
Bangladesh	2	2.6
Egypt	1	1.3
Iraq	1	1.3
Italy	1	1.3
South Africa	1	1.3
West Indies	1	1.3
Not Stated	6	7.8

*training grade of family medicine/general practice see www.mmc.nhs.uk/ for further details.

### Practitioner Language Skills

A total of 23 languages, other than English, were reported as being spoken to some level by the practitioners in the survey, the three most common reflecting the substantial South Asian population of Birmingham (see Table 2). These were Urdu, Hindi and Punjabi. Proficiency in European languages was generally low and no practitioner reported any proficiency in Polish, interesting in the light of the many Poles who have arrived in Birmingham to work since the recent enlargement of the European Union.

**Table 2:** Reported practitioner fluency in other languages spoken.

Language	No proficiency	Basic proficiency	Moderate proficiency	Highly proficient
Urdu	32	9	12	24
Hindi	31	10	13	23
Punjabi	38	14	13	12
Bengali	66	4	3	4
Gujerati	64	7	3	3
Tamil	74	0	0	3
Arabic	70	3	2	2
Telugu	75	0	0	2
Spanish	73	3	0	1
Cantonese	76	0	0	1
Italian	76	0	0	1
Marathi	76	0	0	1
Sindhi	76	0	0	1
Dogri	76	0	0	1
Kurdish	76	0	0	1
British Sign Language	76	0	0	1
French	66	10	1	0
Kanada	75	1	1	0
Malayalam	76	0	1	0
Katchi	76	0	1	0
Mirpuri	76	0	1	0
Swahili	76	1	0	0
German	76	1	0	0

### Patient Characteristics

More female patients were included in the study than male, reflecting the tendency for women to consult than men (see Table 3). Patients were reported as having 38 different first languages other than English. The most common was Urdu (n = 192), closely followed by Punjabi (n = 118) and Bengali (n = 79). Somali was reported to be the first language of 35 patients. A number of other African languages were spoken by individual patients. Two languages were ill-defined as “From Africa” and “Muslim”.

**Table 3:** Demographic and language characteristics of patients seen.

Number of consultations n = 1008	n	%
**Gender**		
Female n (%)	610	60.5
Male	395	39.2
Not specified	3	0.3
**Age**, median (IQR)	35	(20 to 51)
**Consultation time min**, median (IQR)	10	(6 to 13)
**First language**		
English	453	44.9
Urdu	192	19.1
Punjabi	118	11.7
Bengali/Bangla	79	7.8
Somali	35	3.5
Mirpuri	29	2.9
Arabic	26	2.6
Gujerati	15	1.5
Hindi	8	0.8
Hinko	5	0.5
Kurdish	4	0.4
Pushto	4	0.4
Farsi	3	0.3
French	3	0.3
Cantonese	2	0.2
Polish	2	0.2
Portuguese	2	0.2
Shona	2	0.2
Swahili	2	0.2
Telugu	2	0.2

N.B. Eighteen consultations were conducted in languages that were used just once inthe study. These were reported to be Edo, Ezzik, Finnish, Henko, Katchi, Lithuanian, Lunyoro, Malayalam, Mandarin, Marathi, Oriya/Hindi, Patois, Romanian, Spanish, Tamil, Tswana Zulu, Yerba and Yoruba. In 3 cases, the language was either not stated or ill-defined.

### Consultation Language

Consultations took place solely in English in 710 cases (70.4%) and in 7 consultations a combination of English and one other language was reported to have been used (Table 4). 290 consultations were reported as being held in other languages. In 57 of consultations where a language other than English was used, a relative or friend interpreted, while in 6 cases a professional interpreter was used. Five of these cases occurred in the same practice.

**Table 4:** Consultation Language.

	n	%
**English**	710	70.4
**English plus another language** without an interpreter	7	0.7
**Consultation not in English**	290	28.7
Consulted in patient's language without an interpreter*	222	22.0
Relative or friend interpreted*	57	5.7
Professional interpreter used	6	0.6
Bilingual worker or community worker used	6	0.6
**Language Not Stated**	1	0.1

*1 consultation was reported to have taken place in patient's language without an interpreter and that a friend or relative also interpreted, leading it to be counted twice.

Overall, data on practitioners' skills in the language of consultation were provided for 1003 consultations. Practitioners' language skills in the language of consultation are described in Table 5. In 181 consultations conducted in languages other than English, the practitioner stated that they could speak the relevant language moderately well or were highly proficient in it. However, 44 (4.4%) consultations were conducted in a language in which the practitioner reported having no or only basic proficiency (inadequate skills) and where there was no additional person present to interpret. In 21 (2.1%) of these consultations, the practitioner stated that they had no skills in the relevant language.

**Table 5:** Language used in consultation (where other than English).

Consultation language	Practitionerproficiency in language used in consultation:	n	None	Basic	Moderately proficient	Highly proficient
Arabic	Arabic	10	0 (0%)	0 (0%)	0 (0%)	10 (100%)
Bengali	Bengali	40	5 (12.5%)	7 (17.5%)	10 (25.0%)	18 (45.0%)
Bengali/English*	Bengali	4	0 (0%)	4 (100%)	0 (0%)	0 (0%)
Gujerati	Gujerati	3	0 (0%)	1 (33.3%)	0 (0%)	2 (66.7%)
Hindi	Hindi	13	1 (7.7%)	2 (15.4%)	6 (46.2%)	4 (30.8%)
Hindi/Punjabi*	Punjabi	3	0 (0%)	2 (66.7%)	1 (33.3%)	0 (0%)
Katchi	Katchi	1	0 (0%)	0 (0%)	1 (100%)	0 (0%)
Mirpuri	Mirpuri	13	13 (100%)	0 (0%)	0 (0%)	0 (0%)
Punjabi	Punjabi	43	1 (2.3%)	3 (7.0%)	19 (44.2%)	20 (46.5%)
Urdu	Urdu	89	1 (1.1%)	4 (4.5%)	13 (14.6%)	71 (79.8%)
Urdu/English*	Urdu	3	0 (0%)	0 (0%)	0 (0%)	3 (100%)
Urdu/Hindi*	Urdu	2	0 (0%)	0 (0%)	2 (100%)	0(0%)
Urdu/Punjabi*	Urdu	1	0 (0%)	0 (0%)	0 (0%)	1 (100%)
Not stated		4	-	-	-	-
Total		229**	21	23	52	129

*For consultation in two languages, the practitioner was assessed for the first language reported by the patient.

**This total includes 7 consultations conducted in a combination of English and another language.

N.B. This table does not include consultations where a third party was involved in interpreting.

The most common language used was Urdu (89 consultations), followed by Punjabi (43) and Bengali (40). Some consultations were conducted in a combination of two languages.

Exploratory univariate analyses did not demonstrate a relationship between GP age, gender and date of qualification with skills in the language of consultation (Table 6). The relationship of practitioner and patient characteristics with the odds of practitioner adequacy in the language of consultation was examined. It can be seen that no identified characteristic of practitioner or patient was associated with increased or decreased likelihood of the consultation taking place in a language in which the practitioner had inadequate skills.

**Table 6:** Practitioner and patient characteristics and odds of practitioner having inadequate skills in language of consultation – Univariate Analyses.

Characteristic	Odds Ratio	Lower 95% CI	Upper 95% CI	P Value
Family medicine practitioner Age	1.019	0.992	1.047	0.171
Female Practitioner	0.772	0.370	1.609	0.489
Qualification date	0.982	0.954	1.010	0.211
Practitioner - Advanced Nurse Practitioner	1.414	0.284	7.043	0.672
Practitioner – General Practice Principal	0.715	0.309	1.653	0.432
Practitioner – Trainee	1.372	0.360	5.235	0.643
Practitioner - Locum	1.105	0.206	5.938	0.907
Practitioner - Other	0.773	0.172	3.477	0.737
Practitioner - Salaried General Practitioner	1.000			Referent
Patient Age	1.007	0.997	1.018	0.178
Patient Female	1.465	0.916	2.342	0.111

N.B. Odds ratio greater than 1 is associated with inadequate language skills.

## Discussion

The study highlighted a number of issues related to language and healthcare: the range of languages spoken by patients in comparison with those spoken by health practitioners; the strategies used by practitioners to manage language barriers and the number of consultations where basic understanding may be compromised.

The range of languages spoken by patients is unsurprising, given the diversity of people now living in Birmingham. The enlargement of the European Union has meant that many workers have arrived from Eastern Europe, from countries such as Poland, the Czech Republic and Lithuania. In addition, there are asylum seekers and refugees from conflict zones, such as Somalia, Ethiopia, Afghanistan and Zimbabwe. There are also well-established communities originating from the Indian sub-continent, the Caribbean and China. The study suggests that the range of first languages spoken by patients is greater than that spoken by health professionals to any level of proficiency. For example, Somali was reported as being the first language of 35 out 1008 patients in the study, but no health practitioner claimed any proficiency in this language. Furthermore, although 23 languages were identified as being spoken by practitioners, these were not spoken to a high level of proficiency by all those who cited them.

Strategies used by practitioners to overcome language barriers varied. In 57 consultations, family or friends were used to interpret. This is a common strategy in many countries [3]. One Swiss study suggested that 79 per cent of medical and psychiatric staff surveyed often used patients' relatives and friends [19] and a US study reported that 70 per cent of paediatricans surveyed used family members [20]. In the UK, an outpatient department used family members 70 per cent of the time when an interpreter was needed [21]. While this approach has a number of benefits, including saving money on booking professional interpreters and the potential for relatives to offer moral support and to help patients remember complex information, there are well-documented disadvantages [2]. Confidentiality may be compromised and patients may not want to divulge sensitive or intimate information in front of family members or friends. There is also uncertainty about how well the person interpreting can speak the target language, particularly if they are younger family members as is sometimes the case. This can lead to mistakes or incomplete transmission of vital information. Obtaining informed consent may be difficult in such circumstances.

Even more worrying, perhaps, is the number of times where the consultation has been conducted in a language in which the practitioner has declared only basic proficiency or no proficiency at all. This was especially the case for a number of Mirpuri-speaking patients, where in 13 consultations, the practitioner declared no proficiency in that language but reported that the consultation was conducted in Mirpuri (a common language in the HOBtPCT area). Both of the practitioners concerned were proficient in Urdu, so it is possible that there was some mutual understanding. However, there is clearly potential for miscommunication in this situation. Like the tendency to use family interpreters, this is a familiar strategy, called “getting by” in one study, [22] in which physicians described how they “got by” using the few words of a language they knew or even just by using gestures along with physical examination of the patient.

Good practice was also in evidence, both in the use of professional interpreters and in the cases of practitioners being able to communicate effectively in the patient's own language. However, the first strategy was only used six times in the allocated sessions, five of these occurring in the same practice and involving the same practitioner. This is not untypical of what happens internationally. Our own recent systematic review indicates that professional interpreters are often not the preferred option for a number of reasons, including convenience and increased costs [23]. However, patient satisfaction ratings tend to decline when interpreters are not used when patients perceive a need for them [24], [25].

In a substantial number of consultations, the practitioner was able to consult with the patient in their own language, a situation which studies suggest generates highest patient satisfaction ratings. Of the 290 consultations held in languages other than English, there were 181 where the practitioner was able to communicate effectively in the patient's own language (18% of the total number of consultations in this study). The high number of general practitioners who are fluent in languages like Punjabi. Urdu, Hindi and Arabic makes this possible and this evidently saves the health authority a considerable amount of money that would otherwise have been needed for professional interpreters. There was evidence to suggest that patients actively seek out family physicians who speak their language, for example, in one session where 15 patients were seen, ten consultations were conducted in Arabic.

Based on our findings we estimate that if professional interpreters were used for all of those consultations where a friend or relative interpreted or where the practitioner conducted the consultation in a language in which they reported inadequate skills, that this would cost the health authority an extra £2 million pounds per year [23].

### Strengths and limitations

Firstly, there was a high (63%) response rate from professionals, which is more remarkable given that this survey was undertaken during the Swine flu epidemic in 2009. It is not known whether non-responders differed in any way from those who did. For example, it is possible that those practitioners who were most interested in language diversity or who had multilingual patient lists would be more likely to participate. However, the list of all family medicine professionals in HOBtPCT was examined and there appeared to be no significant differences in characteristics such as age, gender or ethnicity. Secondly, practitioners made a subjective assessment of the English proficiency of patients and ideas of proficiency may have differed between practitioners. Thirdly, practitioners made a subjective assessment of their own proficiency in specific languages and, again, what one person might regard as speaking a language moderately well another might regard as a basic proficiency.

Finally, this study was conducted in just one PCT and policies and procedures may well be different in other PCTs.

### Conclusions

It would appear that professional interpreters are under-used in relation to the need for them, with bilingual staff or family and friends being used in many cases. In a substantial number of cases where the patient spoke little or no English, the practitioner consulted in the patient's language but this approach was also used where reported practitioner proficiency was low.

More research is needed on what happens in those consultations where the practitioner has attempted to consult in a language in which s/he is not proficient or where family or friends are used to interpret. The reasons for health professionals failing to use professional interpreters where there are language constraints could also be usefully researched. Finally, the costs, both health-related and economic (unnecessary tests, repeat appointments, non-compliance), of not using professional interpreters where needed should be investigated.

There is a need for provision of interpreters in the UK NHS as it is not mandatory; unlike the US [5] as clinicians are ultimately responsible for ensuring effective communication with their patients in improving patient care and safety.

## Supporting Information

Appendix S1 **Questionnaire.**
(DOC)
