# Supplementary material for: The Unmet Need for Interpreting Provision in UK Primary Care
Source: PLoS One. 2011 Jun 13;6(6):e20837. doi: 10.1371/journal.pone.0020837 (PMC3113854; doi:10.1371/journal.pone.0020837)
Supplement: Appendix S1 — Questionnaire. (DOC) [file pone.0020837.s001.doc]

**Appendix S1: Questionnaire**

**Modelling the possible need for and provision of interpreters in primary care**

***Form 1* Practitioner Details**

**Name:**

***Job Title:*** (Please tick box below appropriate description)

| GP Principal | Salaried GP | GP Trainee | Locum | Advanced Nurse Practitioner | Other  *(please state)* |
| --- | --- | --- | --- | --- | --- |
|  |  |  |  |  |  |

**Practice:**

**Post Code of Practice:**

**Date of session being recorded:**

**What languages do you speak, in addition to English? Please tick as appropriate, indicating your own proficiency**

| **Language** | **Basic** | **Moderately well** | **Highly proficient** |
| --- | --- | --- | --- |
| Cantonese |  |  |  |
| French |  |  |  |
| Gujerati |  |  |  |
| Hindi |  |  |  |
| Mandarin |  |  |  |
| Polish |  |  |  |
| Punjabi |  |  |  |
| Spanish |  |  |  |
| Somali |  |  |  |
| Urdu |  |  |  |
| British Sign Language |  |  |  |
| Other (please state which) |  |  |  |
|  |  |  |  |
|  |  |  |  |
|  |  |  |  |
|  |  |  |  |

**Thank you for taking part in this survey. Please hand this form together with the completed Consultation Record Sheet and Consent Form to your Practice Manager at the end of the session.**

***Form 2* Consultation Record Sheet**

This form is designed to help you record the language needs of your patients. Please input details of all patients you see in your designated session, whether or not they are fluent English speakers. See completed example and information sheet for additional help.

| Consultation | | Patient details | | | | Consult-  ation  took place in English  Please tick | You consulted in patient’s language without an interpreter  Please tick and state language | Relative or friend interpreted  Please tick | Prof.  interpreter  used  Please insert P if physically present,  T if by telephone,  S if signer used | Other  Please insert B if bilingual staff member or C if commun-ity worker | Consultation abandoned because of language difficulties  Please tick |
| --- | --- | --- | --- | --- | --- | --- | --- | --- | --- | --- | --- |
| Start | End | M/F | Age | First  language | Profici-ency * |  |  |  |  |  |  |
|  |  |  |  |  | 0 1 2 3 |  |  |  |  |  |  |
|  |  |  |  |  | 0 1 2 3 |  |  |  |  |  |  |
|  |  |  |  |  | 0 1 2 3 |  |  |  |  |  |  |
|  |  |  |  |  | 0 1 2 3 |  |  |  |  |  |  |
|  |  |  |  |  | 0 1 2 3 |  |  |  |  |  |  |
|  |  |  |  |  | 0 1 2 3 |  |  |  |  |  |  |
|  |  |  |  |  | 0 1 2 3 |  |  |  |  |  |  |
|  |  |  |  |  | 0 1 2 3 |  |  |  |  |  |  |
|  |  |  |  |  | 0 1 2 3 |  |  |  |  |  |  |
|  |  |  |  |  | 0 1 2 3 |  |  |  |  |  |  |
|  |  |  |  |  | 0 1 2 3 |  |  |  |  |  |  |
|  |  |  |  |  | 0 1 2 3 |  |  |  |  |  |  |
|  |  |  |  |  | 0 1 2 3 |  |  |  |  |  |  |
|  |  |  |  |  | 0 1 2 3 |  |  |  |  |  |  |
|  |  |  |  |  | 0 1 2 3 |  |  |  |  |  |  |
|  |  |  |  |  | 0 1 2 3 |  |  |  |  |  |  |
|  |  |  |  |  | 0 1 2 3 |  |  |  |  |  |  |
|  |  |  |  |  | 0 1 2 3 |  |  |  |  |  |  |
|  |  |  |  |  | 0 1 2 3 |  |  |  |  |  |  |
|  |  |  |  |  | 0 1 2 3 |  |  |  |  |  |  |
|  |  |  |  |  | 0 1 2 3 |  |  |  |  |  |  |
|  |  |  |  |  | 0 1 2 3 |  |  |  |  |  |  |
|  |  |  |  |  | 0 1 2 3 |  |  |  |  |  |  |
|  |  |  |  |  | 0 1 2 3 |  |  |  |  |  |  |
|  |  |  |  |  | 0 1 2 3 |  |  |  |  |  |  |
|  |  |  |  |  | 0 1 2 3 |  |  |  |  |  |  |
|  |  |  |  |  | 0 1 2 3 |  |  |  |  |  |  |
|  |  |  |  |  | 0 1 2 3 |  |  |  |  |  |  |
|  |  |  |  |  | 0 1 2 3 |  |  |  |  |  |  |

* Proficiency Scores: 0 = none; 1 = basic; 2 = moderate; 3 = high (Please underline the appropriate number)

**Thank you for taking part in this survey. When completed, please hand this form together with the completed Practitioner Details front sheet and Consent Form to your Practice Manager at the end of the session.**
